# Supplementary material for: Determinants of maternal influenza vaccination in the context of low- and middle-income countries: A systematic review
Source: PLoS One. 2022 Jan 26;17(1):e0262871. doi: 10.1371/journal.pone.0262871 (PMC8791521; doi:10.1371/journal.pone.0262871)
Supplement: S1 Table — (DOCX) [file pone.0262871.s001.docx]

**Supplementary Table 1: Quality assessment of studies using New Castle Ottawa Scale**

| **Author** | **Year** | **Selection** | | | | **Comparability** | **Outcome** | | **Score** | **Quality** |
| --- | --- | --- | --- | --- | --- | --- | --- | --- | --- | --- |
|  |  | **Representativeness of the Sample** | **Sample size** | **Ascertainment of the exposure** | **Non respondents** | **The subjects in different outcomes are comparable** | **Assessment of the outcome** | **Statistical tests** |  |  |
| Arriola | 2018 | * | * | * | * | ** | * | * | 8 | Low risk of bias |
| Khan | 2015 | * | 0 | 0 | * | * | * | * | 5 | High risk of bias |
| Reinders | 2019 | * | * | * | * | ** | * | * | 8 | Low risk of bias |
| Armitage | 2018 | * | * | * | * | ** | * | * | 8 | Low risk of bias |
| Arriola | 2015 | * | * | * | * | ** | * | * | 8 | Low risk of bias |
| Koul | 2014 | 0 | 0 | * | * | 0 | * | 0 | 3 | Very high risk of bias |
| Fleming | 2018 | * | 0 | 0 | 0 | 0 | * | 0 | 2 | Very high risk of bias |
| Kouassi | 2012 | * | * | * | * | ** | * | * | 8 | Low risk of bias |
| Fleming | 2018 | * | 0 | 0 | 0 | 0 | * | 0 | 2 | Very high risk of bias |

Low risk of bias=7-9 , high risk of bias=4-6, very high risk of bias= 0-3 [Ref: BMC Medical Research Methodology 2014, 14:45 http://www.biomedcentral.com/1471-2288/14/45]
