# Supplementary material for: Determinants of maternal influenza vaccination in the context of low- and middle-income countries: A systematic review
Source: PLoS One. 2022 Jan 26;17(1):e0262871. doi: 10.1371/journal.pone.0262871 (PMC8791521; doi:10.1371/journal.pone.0262871)
Supplement: S2 Table — (DOCX) [file pone.0262871.s002.docx]

**Supplementary Table2 : Quality assessment of qualitative studies using tool developed by Hawker et al.**

| **Author** | **Year** | **Abstract and title** | **Introduction**  **and Aims** | **Methods and data** | **Sampling** | **Data analysis** | **Ethics and bias** | **Results** | **Transferability /**  **Generalisability** | **Implications / usefulness** | **Score** |
| --- | --- | --- | --- | --- | --- | --- | --- | --- | --- | --- | --- |
| Top | 2018 | 4 | 3 | 2 | 2 | 2 | 2 | 3 | 2 | 3 | Low quality |
| Lohiniva | 2014 | 3 | 1 | 3 | 3 | 3 | 4 | 3 | 4 | 4 | Medium quality |

30-36 = high quality, 24-29= medium quality, 9-24=low quality [Ref: Qualitative health research. 2002 Nov;12(9):1284–99].
