# Supplementary material for: Determinants of maternal influenza vaccination in the context of low- and middle-income countries: A systematic review
Source: PLoS One. 2022 Jan 26;17(1):e0262871. doi: 10.1371/journal.pone.0262871 (PMC8791521; doi:10.1371/journal.pone.0262871)
Supplement: Attachment — Submitted filename: Response to reviewers_Mat flu SR_05102021.docx [file pone.0262871.s003.docx]

**Response to reviewers**

Throughout the document, please pay attention and clarify to whenever “accept” and “acceptance” are used versus “uptake”. In many places (as I point out) it seems as if the authors equate acceptance to uptake, which eclipses the fact that low uptake may be influenced by access issues and systemic factors (that are independent of acceptance). A woman cannot accept a vaccine she is never offered, and it is unclear throughout the review if the factors and rates identified are within the context of women being offered the vaccine or in real-world situations where the vaccine may not be available. Some additional commentary about this issue (whether the main problem is acceptance or availability) would strengthen the discussion.
I also suggest making it more clear in Table 3 whether each factor identified relates to the outcome of acceptance, uptake or intention. These are all quite different and indicating the precise outcome would help the reader better interpret the ORs and RR reported in the right-hand column. This could be done by a symbol or an additional column.

Thank you very much for this constructive feedback. We have now used appropriate terminologies (acceptance or uptake) in the revised text. The table 3 is also revised accordingly.

**Abstract:**
- Please clarify what you mean by “barriers amongst health care providers” in the abstract. As it is written now, it is unclear if these are “barriers that inhibit healthcare providers from offering the vaccine to pregnant women”, or simply “barriers to vaccination as perceived by healthcare providers”.

These are the barriers that inhibit the health care providers from offering the vaccine to pregnant women. This has been clarified in the abstract now.

- Your concluding statement identifies steps to improve “acceptance” of maternal influenza vaccine, suggesting that identifying barriers and facilitators to “acceptance” was the main goal of the study. However, this in not specified in the aim as written in the background. Barriers and facilitators can be multilevel, and include systemic barriers (supply, availability) that have nothing to do with acceptance. Please clarify in your aims and conclusion statement if your review was aimed at identifying barriers and facilitators specifically to acceptance or more broadly, to uptake.

Thank you for this comment. As you have pointed out, we aim to review the uptake of maternal influenza vaccine broadly which includes acceptance wherever applicable. We have clarified this point in the revised aims and conclusions.

**Background**
- Second sentence: Please clarify what population this hospitalization rate applies to (e.g. India?, low-and-middle income countries? Global?)

These are global hospitalisation rates for influenza related hospitalisation. We have revised the sentence as follows:

“Globally, the influenza-related hospitalisation rate in pregnant women is estimated to be 42.1% (interquartile range (IQR), 22.5-60.4%) and among them around 8% (IQR, 5.9-12.7%) have severe disease that results in intensive care admission or death(4).”

- Second paragraph: Beginning with the second sentence, please specify that the literature reviewed pertains to research globally (including high income countries) so that the reader does not misinterpret that this research is specific to LMICs

The text pertains to global literature on maternal influenza. We have specified this in the revised text now.

- Second paragraph, Last sentence: acceptability among who? Women? HCPs? Stakeholders? Also, why is it important to examine acceptability specifically versus other barriers? (see comment in abstract)

We have revised this sentence as follows:

“This systematic review was planned to synthesize evidence about barriers and facilitators for maternal influenza vaccination amongst HCPs and pregnant women in LMICs.”

**Methods**
- “till” is not a full word. Please replace with “until” or “up until”

We have revised the sentence as follows:

“We included studies published until Dec 31, 2020 amongst women in the antenatal as well as postnatal period.”

- Please capitalize “World Bank”

The text has been revised accordingly.

**Results**
- There are a few articles that are classified as having very high risk of bias. How did this work into your overall interpretation of results?

Thank you. We have addressed this in the revised text in discussion:

“Four out of 11 studies had high or very high risk of bias. This is one of the serious limitations of this review which reduces the confidence in the results from these studies.”

- Please capitalize Coast” (as in “Ivory Coast”)

The text has been revised accordingly.

- A more appropriate subheading would be “Vaccination rate” instead of “vaccination acceptance”. The term “acceptance” implies that all women were offered the vaccine and only some “accepted” and this might not be the case. If you do mean acceptance, a sentence is warranted about the background of the study explaining why this outcome is possible to measured (e.g. ensuring that all women in the denominator indeed had access to the vaccine).

We would like to clarify that ref 25, 27 and 28 report vaccination rate or uptake and ref 23, 24, 29 report willingness to get vaccinated if the vaccine is made available. To include both these scenarios, we have changed the heading to “Vaccine acceptance or vaccine uptake” in the revised text.

- Similarly, “Women with higher education were 36-42% less likely to receive or accept influenza…” In this sentence, which do you mean, receive, or accept? They have quite different meanings.

The verb accept would be more appropriate in this case, as the influenza vaccination was provided under government program in Peru and was available to all free of cost. We have made the required change.

- Similarly, I would not use the word “willing” as in “Women were more willing to receive” if non-availability is really a factor. Women could have been willing but unable to receive it because of non-availability. Using the word “willing” places all responsibility/blame on the woman when lack of uptake of the vaccine could be due to non-availability, independent of her willingness to receive it.

The term willingness has been used for three studies reported from Pakistan (not in immunisation program but recommended to pregnant women), Ivory coast (vaccine not free but responses of the participants were recorded for their intent to get vaccinated) and Gambia (vaccine was under clinical trial mode and participants were asked about their willingness / intent for use in pregnancy if made available). As these studies have investigated the willingness or intent to get vaccinated, the word ‘willingness’ is appropriate here.

- In the first sentence of “Factors related to healthcare providers and health system” please specify, the odds of receiving the vaccine was higher when recommended by a HCP compared to what? When an HCP recommends against the vaccine? When someone other than the HCP recommends the vaccine? When an HCP offers no opinion on the vaccine? What was the comparison group here?

The comparison group here was women who did not receive any recommendation from their health care providers. We have modified the sentence as follows:

“The odds of receiving influenza vaccine were reported 2.5 to 74 times higher in pregnant women who received a recommendation from an HCP as compared to those who did not receive any recommendation (30)(27)(26).”

- Do you really mean “Lack of respect toward the HCP” or “Lack of respect by the HCP (toward the patient)?” Please clarify.

Thank you for this comment. We have clarified the term as “lack of respect by HCPs towards confidentiality of the participants” in the revised text.

**Discussion**
- Again, ensure and clarify that whenever the word “acceptance” is used, it means a vaccine was available to all women and they had the opportunity to accept.

Thank you. We have now used the word acceptance in the discussion only where it is appropriate.

- I advise caution extrapolating the lower rate of vaccination in pregnancy vs. other population in reference 36 to vaccine hesitancy without sufficient supporting evidence. There may be other systemic factors that explain this lower rate.

We have revised the text as follows:

“However, the coverage for influenza vaccination was lower during pregnancy than other populations (e.g. children and elderly) in Peru and Nicaragua(36). These findings are similar to other global data indicating low uptake of influenza vaccine uptake during pregnancy(37)”

- Does the evidence point to acceptance or access as the biggest barrier to vaccination during pregnancy in LMIC? Can you make a determination or comment on what the literature suggests, or point out the need for research to address this gap?

Our evidence from Peru study points out that acceptance may play equally important role. To address this, we have added the following sentence in the discussion:

“However, as pointed out in the Peru study (25), the vaccine uptake may remain low despite subsidized vaccine program. This highlights the importance of including awareness campaigns for general public and health care providers in order to improve vaccine coverage.”

However, we have a limitation that only three of the included studies had vaccination included in the health programs and the other studies only assessed willingness to get vaccination in the absence of actual program. Hence, we have added following line as a limitation in the discussion:

“Except Nicaragua (27) (28) and Peru (25), none of the countries had maternal influenza vaccination included in the health program. Hence, there was limited information available about the logistic or operational factors related to availability of influenza vaccine for pregnant women in these countries, which can play very important role in the success of implementation.

“lack of research focusing on the policy makers” ◊ I believe “on” is missing

Thank you for pointing out this typographical error. We have corrected the mistake in the revised text.

I am surprised that “improving health literacy” is the first point that you bring forth in your conclusion. I don’t believe that the review as a whole point to health literacy as the main issue. Please be more comprehensive in your conclusion to point out the myriad of multilevel factors that must be addressed to improve uptake (as well as acceptance).

Thank you for your feedback. The conclusion is modified accordingly. The revised conclusion is as follows:

“Higher educational status, better access to antenatal care, perceived risk of influenza during pregnancy, perceived benefits of influenza vaccination during pregnancy, recommendation by health care providers and inclusion of maternal influenza vaccine in health policy were important facilitators for maternal influenza vaccine uptake in LMIC. Fear of adverse effects, uncertainty about the benefits of vaccination and ineffective health communication regarding the influenza vaccine were barriers identified.

Thus, while availability of influenza vaccine in public health system can be a key to the success of vaccine implementation program, increasing the public health awareness about need and benefits of maternal influenza vaccination amongst pregnant women as well as their health care providers is crucial to improve the acceptance of maternal influenza vaccination in low and middle-income countries.”

Thank you for pointing out the lack of research on this issue. I do hope your article inspires more research on this important topic.

Thank you for your feedback. We do hope the same.

**Table** **3**

- Unless the number of antenatal visits reflects official recommendation/policy, I would put this factor under demographic/ clinical factors (not health systems).

The minimum number of antenatal visits at both public and private health care setup for a healthy pregnancy is decided by a health program in the country. Hence, we argue that number of antenatal visits should be part of health system.
