# Supplementary material for: Determinants of maternal influenza vaccination in the context of low- and middle-income countries: A systematic review
Source: PLoS One. 2022 Jan 26;17(1):e0262871. doi: 10.1371/journal.pone.0262871 (PMC8791521; doi:10.1371/journal.pone.0262871)
Supplement: Attachment — Submitted filename: Response to reviewers23112021.docx [file pone.0262871.s004.docx]

Additional Editor Comments (if provided):

- The addition of the final column in Table 3 is helpful in understanding the outcome of each study. In the "Vaccine acceptance and vaccine uptake" section of the manuscript, before you introduce Table 3, it might be helpful to orient the reader to this column these terms by defining them. For example, you might say "Outcomes in the studies included overall vaccine uptake as well as vaccine acceptance (patient agreement to vaccine assuming it was offered) (Table 3)."

Thank you for this valuable input. We have added this sentence under vaccine acceptance and vaccine uptake.

- "uptake" might also be added to the title of table 3 ".. uptake and acceptance of..."

Thank you. We have made the required changes.

- Please clarify what you mean by "clubbing". Do you mean "combining"?

We clarified this by replacing the term clubbing with combining.

- I agree that variable of number of antenatal visits, if it purely reflects a policy, should remain under health systems factors. Would be appropriate to label this variable "Recommended number of antenatal visits" so that the reader knows this reflects the ideal number as set by national recommendations? If not, it might still fit better under "clinical factors". Similar to the variable "vaccination in third trimester", number of antenatal visits is determined by both the individual and the health system (as even if 4 visits are recommended, many women will not complete that amount).
Agree with this. We have added antenatal visits under clinical factors.

[Note: HTML markup is below. Please do not edit.]

Reviewers' comments:

Reviewer's Responses to Questions

**Comments to the Author**

1. If the authors have adequately addressed your comments raised in a previous round of review and you feel that this manuscript is now acceptable for publication, you may indicate that here to bypass the “Comments to the Author” section, enter your conflict of interest statement in the “Confidential to Editor” section, and submit your "Accept" recommendation.

Reviewer #1: All comments have been addressed

2. Is the manuscript technically sound, and do the data support the conclusions?

The manuscript must describe a technically sound piece of scientific research with data that supports the conclusions. Experiments must have been conducted rigorously, with appropriate controls, replication, and sample sizes. The conclusions must be drawn appropriately based on the data presented.

Reviewer #1: Yes

3. Has the statistical analysis been performed appropriately and rigorously?

Reviewer #1: Yes

4. Have the authors made all data underlying the findings in their manuscript fully available?

The [PLOS Data policy](http://www.plosone.org/static/policies.action#sharing) requires authors to make all data underlying the findings described in their manuscript fully available without restriction, with rare exception (please refer to the Data Availability Statement in the manuscript PDF file). The data should be provided as part of the manuscript or its supporting information, or deposited to a public repository. For example, in addition to summary statistics, the data points behind means, medians and variance measures should be available. If there are restrictions on publicly sharing data—e.g. participant privacy or use of data from a third party—those must be specified.

Reviewer #1: Yes

5. Is the manuscript presented in an intelligible fashion and written in standard English?

PLOS ONE does not copyedit accepted manuscripts, so the language in submitted articles must be clear, correct, and unambiguous. Any typographical or grammatical errors should be corrected at revision, so please note any specific errors here.

Reviewer #1: Yes

6. Review Comments to the Author

Please use the space provided to explain your answers to the questions above. You may also include additional comments for the author, including concerns about dual publication, research ethics, or publication ethics. (Please upload your review as an attachment if it exceeds 20,000 characters)

Reviewer #1: The revised manuscript has addressed most of the issues raised by reviewers.

The suggestion "This review can be improved by including the reasons by rejecting large number of publications (including 19 reviews). Has this huge rejection introduced any bias, needs to be explained. If there is any way of analyzing these rejected 19 reviews and summarize findings from these reviews in a paragraph or table, it might be a way of minimizing impact of rejecting valid reviews" has not been addressed adequately though.

We have added the following text at the end of the discussion:

“We have excluded full text articles from high income countries and focused on the studies from LMIC only. This is because the problems faced by LMIC are likely to be different from higher income countries due to differences in the socioeconomic status, literacy and health care access for pregnant women. However, we have discussed the findings from these studies at relevant places in the introduction and discussion.”

We have now included all the relevant reviews (listed below) from our excluded list of articles in the background discussion.

1. Hirve S, Lambach P, Paget J, Vandemaele K, Fitzner J, Zhang W. Seasonal influenza vaccine policy, use and effectiveness in the tropics and subtropics - a systematic literature review. Influenza and other respiratory viruses. 2016 Jul;10(4):254–67

2. Regan A, Haberg S, Fell DB. Current Perspectives on Maternal Influenza Immunization. Current Tropical Medicine Reports. 2019;6(4):239–49.

3. Lutz CS, Carr W, Cohn A, Rodriguez L. Understanding barriers and predictors of maternal immunization: Identifying gaps through an exploratory literature review. Vaccine. 2018 Nov;36(49):7445–55.

4. Morales KF, Menning L, Lambach P. The faces of influenza vaccine recommendation: A Literature review of the determinants and barriers to health providers’ recommendation of influenza vaccine in pregnancy. Vaccine. 2020 Jun;38(31):4805–15.

5. Buchy P, Badur S, Kassianos G, Preiss S, Tam JS, P. B, et al. Vaccinating pregnant women against influenza needs to be a priority for all countries: An expert commentary. International Journal of Infectious Diseases 2020 Mar;92:1–12.

6. Yuen CYS, Tarrant M. Determinants of uptake of influenza vaccination among pregnant women - a systematic review. Vaccine. 2014 Aug;32(36):4602–13.

7. Yuen CYS, Tarrant M. A comprehensive review of influenza and influenza vaccination during pregnancy. The Journal of perinatal & neonatal nursing. 2014;28(4):261–70.

8. Wong VWY, Lok KYW, Tarrant M. Interventions to increase the uptake of seasonal influenza vaccination among pregnant women: A systematic review. Vaccine. 2016 Jan;34(1):20–32.

9. Raya BA, Edwards KM, Scheifele DW, Halperin SA, B. AR, K.M. E, et al. Pertussis and influenza immunisation during pregnancy: a landscape review. The Lancet Infectious Diseases. 2017 Jul;17(7):e209–22.

10. Phadke VK, Omer SB. Maternal vaccination for the prevention of influenza: current status and hopes for the future. Expert Review of Vaccines 2016 Oct;15(10):1255–80

The reference numbers 32 and 34 are repeat, need to check and revise the numbers.

Thank you for pointing this out. We have removed the duplicate reference.

The modified manuscript can be accepted as it's a valuable contribution from LMICs to the field.

7. PLOS authors have the option to publish the peer review history of their article ([what does this mean?](https://journals.plos.org/plosone/s/editorial-and-peer-review-process#loc-peer-review-history)). If published, this will include your full peer review and any attached files.


If you choose “no”, your identity will remain anonymous but your review may still be made public.


**Do you want your identity to be public for this peer review?** For information about this choice, including consent withdrawal, please see our [Privacy Policy](https://www.plos.org/privacy-policy).

Reviewer #1: No
